# Protocol to assess retinal metabolic flux of mice via stable isotope-resolved metabolomics

**DOI:** 10.1016/j.xpro.2025.104252

**Published:** 2025-12-27

**Authors:** Georgy Komissarov, Kriti Pandey, Nicholas D. Nolan, Thomas Winogrodzki, Daniel T. Hass, Aykut Demirkol, Brian M. Robbings, James B. Hurley, Stephen H. Tsang

**Affiliations:** 1 Harkness Eye Institute Coordinating Center, Jonas Children’s Vision Care and Barbara & Donald Jonas Stem Cell Laboratory, Institute of Human Nutrition, Columbia Stem Cell Initiative, New York, NY 10032, USA; 2 Department of Biochemistry, University of Washington, Seattle, WA 98195, USA; 3 Department of Biomedical Engineering, Columbia University, New York, NY 10027, USA; 4 Department of Ophthalmology, Columbia University Irving Medical Center, New York, NY 10032, USA; 5 Department of Laboratory Medicine and Pathology, University of Washington School of Medicine, Seattle, WA 98195, USA; 6 Department of Ophthalmology, University of Washington, Seattle, WA 98195, USA; 7 Vagelos College of Physicians and Surgeons, Department of Pathology & Cell Biology, Columbia University Irving Medical Center, New York, NY 10032, USA

**Keywords:** Metabolism, Metabolomics, Mass spectrometry

## Abstract

Here, we present a protocol for evaluating glucose metabolism in mouse retinas and retinal pigment epithelium (RPE)-choroid tissue by tracking the incorporation of ^13^C_6_ from U-^13^C_6_-glucose with gas chromatography-mass spectrometry (GC-MS). We describe steps for incubating tissues in Krebs-Ringer bicarbonate solution and homogenizing tissues. We then detail procedures for extracting metabolites and determining isotopic labeling of intermediates in glycolysis and the tricarboxylic acid (TCA) cycle using GC-MS. The approach has been adapted to study glucose metabolism in various tissues, animal models, and genetic conditions.

For complete details on the use and execution of this protocol, please refer to Nolan et al.^1^

## Highlights

• Guidelines for dissecting mouse retina and RPE-choroid
• Steps for incubating tissues with ^13^C-glucose
• Instructions for extracting metabolites after incubation
• Procedure for analyzing isotopic enrichment by GC-MS

## Abstract (editor highlights)

Publisher’s note: Undertaking any experimental protocol requires adherence to local institutional guidelines for laboratory safety and ethics.

## Graphical abstract

**Figure:**
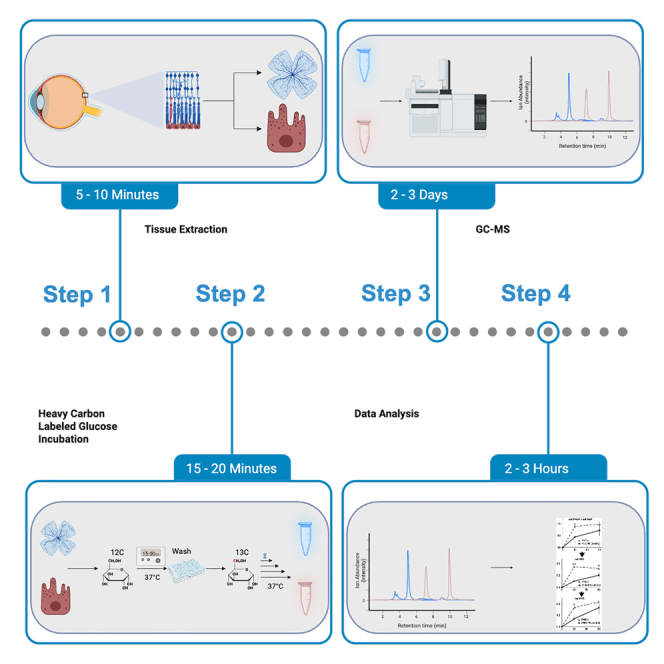

## Teaser

Here, we present a protocol for evaluating glucose metabolism in mouse retinas and RPE-choroid tissue by tracking the incorporation of ^13^C_6_ from U-^13^C_6_-glucose with gas chromatography-mass spectrometry (GC-MS). We describe steps for incubating tissues in Krebs-Ringer bicarbonate solution and homogenizing tissues. We then detail procedures for extracting metabolites and determining isotopic labeling of intermediates in glycolysis and the tricarboxylic acid (TCA) cycle using GC-MS. The approach has been adapted to study glucose metabolism in various tissues, animal models, and genetic conditions.

## Before you begin

Changes to retinal cellular metabolism are an emerging hallmark associated with retinal dystrophies such as retinitis pigmentosa. Isotope tracing and metabolomics have made pinpointing the potential sites of dysfunction more accessible than ever. The following protocol describes the steps for analyzing glycolytic and TCA cycle activity in retinal-degenerate mouse retina and RPE-choroid tissue.^1^ Similar protocols can and have been adapted for use in other tissues and cells. For other metabolic processes, some redesigning has to be done with regard to which isotope-labeled metabolite is used for tissue incubation, as well as which metabolites to help analyze the metabolic pathway of interest.

All animal experiments were conducted as approved by the Columbia University Institutional Animal Care and Use Committee. This protocol utilizes a 37°C incubator with 5% CO_2_ for tissue incubations.

### Institutional permissions

All animal studies were conducted according to the Institutional Animal Care and Use Committee Protocol Number AABU2668 at Columbia University Irving Medical Center. Proper regulatory approval must be obtained before performing the protocol using live vertebrate animals and tissues.

### Making solutions and preparing plates

**Timing: 75–90 min**

1. Prepare Krebs-Ringer-Bicarbonate (KRB) buffer.
  a. Make a solution with the following reagents and molarity.
    i. NaCl (98.5 mM), KCl (4.9 mM), KH_2_PO_4_ (1.2 mM), MgSO_4_ · 7H_2_O (1.2 mM), HEPES (20 mM), CaCl · 2H_2_O (2.6 mM), NaHCO_3_ (25.9 mM).
    ii. Prepare at least 100μL of buffer for each eye, totaling 2 mL for 20 eyes.
    iii. Example: In 500 ml of milli-Q H_2_O mix 2.998 grams of NaCl, 0.1905 grams of KCl, 0.085 grams of KH_2_PO_4_, 0.154 grams of MgSO_4_ · 7H_2_O, 2.4825 grams of HEPES, 0.199 grams of CaCl · 2H_2_O, 1.1335 grams of NaHCO_3_. Stir until dissolved.
  b. After all solutes are dissolved, filter the solution through a 0.22 μm filter.
    i. Aliquot the necessary volume for the samples, according to step 1.a.ii.
  c. Equilibrate the pH of the KRB stock by placing it into a petri dish or well plate in a 37 ^o^C incubator with 5% CO_2_ for 30 minutes.

***Note:*** For a more detailed formulation, including masses/volumes, please see the materials and equipment section.

***Note:*** Unused buffer stock should be stored in a tightly closed storage bottle container at 4°C when not in use for up to 24 months.

2. Prepare sterilised 500 mM stocks of D-Glucose (U-^12^C and U-^13^C_6_).
  a. Prepare a 500 mM stock solution using D-Glucose U-^13^C_6_.
    i. Example: Dissolve 15.0 mg in 161.1 μl ddH_2_O.
  b. Prepare a 500 mM stock solution using D-Glucose U-^12^C.
    i. Example: Dissolve 14.7 mg in 163.2 μl ddH_2_O.

***Note:*** Using higher amounts of D-Glucose stock allows the avoidance of low-weight scale inaccuracies. Aliquots of the solutions and their dilutions can be stored in −80°C for up to 18 months.

3. Prepare 5 mM dilution of D-Glucose U-^12^C and D-Glucose U-^13^C_6_ stock solutions in KRB buffer prepared in step 1.c.
  a. Prepare 5 mM dilution - referred to as 13C:
    i. Example: Dilute 10 μl of 500 mM D-Glucose U-^13^C_6_ solution in 960 μl of KRB buffer and 30 μl of milli-Q-grade ddH_2_O for a total volume of 1 mL.
  b. Prepare 5 mM dilution - referred to as 12C:
    i. Example: Dilute 10μl of 500 mM D-Glucose U-^12^C solution in 960 μl of KRB buffer and 30 μl of milli-Q-grade ddH_2_O for a total volume of 1 mL.

***Note:*** 100 μL/tissue type is used in this protocol for a 96-well incubation plate.

***Note:*** These examples (3.a.i and 3.b.i) are calculated for 1 mL, a sufficient quantity for 9 eyes (with extra buffer to account for pipetting error). Adjust the dilution to suit your intended sample size.

4. Prepare well plates and incubators.
  a. Prepare a 24-well wash plate by adding 1 ml of Gibco HBSS to at least 3 wells per tissue sample.
    i. One well should be used after dissection.
    ii. One well should be used after 12C incubation.
    iii. One well should be used after 13C incubation.
  b. Prepare a 96-well incubation plate by adding 100 μl of 12C and 100 μl 13C into separate wells.
    i. Make 1 well of 12C and 1 well of 13C per tissue for each eyeball.
  c. Place the 96-well incubation plate into an incubator (37°C, 5% CO_2_) for at least 10–15 minutes for equilibration before the start of experimentation.

**CRITICAL:** Do not use the same well for HBSS washing or incubating twice, especially after 12°C and 13°C incubation.

**Table 1:** Metabolites used in standard mix for GC-MS analysis, along with their retention time, molecular mass and M-57 ion

Metabolite	Retention time (minutes)	Molecular mass(g/mol)	M-57 ion
Methylsuccinate	20.29	132.11	303
Palmitate	30.01	256.43	329
Lactate	14.03	90.08	261
Pyruvate	8.21	88.06	174
dihydroxyacetone phosphate	31.34	170.06	484
3-Phosphoglycerate	35.43	186.06	585
Phosphoenol pyruvate	29.09	168.04	453
Citrate	35.24	192.12	591
Isocitrate	35.43	192.12	591
alpha-ketoglutarate	25.15	146.11	346
Succinate	20.09	118.09	289
Fumarate	19.73	116.07	287
Malate	26.97	134.08	419
Alanine	15.13	89.09	260
Serine	24.70	105.09	390
Glycine	15.69	75.07	246
Aspartate	27.72	133.10	418
Glutamate	29.74	147.13	432
Glutamine	32.23	146.14	431
Beta-Hydroxybutyrate	16.46	104.10	275

M-57 ion is the fragment that is used to identify and quantify intensity of the metabolite. These metabolites are involved in various metabolic pathways including glycolysis, TCA cycle, amino acid metabolism and lipid/fatty acid metabolism.^2^

### Metabolic analysis preparation

**Timing: 3–4 h**

5. Prepare a standard mix of metabolites in Table 1, as in Du et al.^2^
  a. This mix is made from known concentrations of solid metabolites dissolved in HPLC-grade water. A 50 μM solution is prepared, 50 μM of each metabolite we routinely monitor (listed in Table 1). We dry aliquots of this solution and store them at −80°C.

6. Prepare the metabolite extraction buffer and standard curve dilution.
  a. Fill a bucket with dry ice and place a metal block on it to cool.
  b. Remove samples from the −80°C freezer, and place them on a metal block.
  c. Thaw the ‘standard mix’.
  d. Prepare standard curve dilutions.
    i. Pipette 20, 10, 4, 2, and 0.4 μL of the ‘standard mix’ into individual 1.5 mL Eppendorf tubes. These volumes and concentrations generate a standard curve consisting of 0, 0.02, 0.1, 0.2, 0.5, and 1 nmol of each metabolic intermediate being studied.
  e. Prepare 10 mM methylsuccinate solution.
    i. Example: Add 1.3211 mg methylsuccinate in 1 mL HPLC-grade H_2_O.
      ***Note:*** The prepared solution can be aliquoted and stored at −20°C for 6 months or −80°C for a year.
  f. Prepare the metabolite extraction buffer.
    i. Prepare 150 μL of the extraction buffer for each sample.
    ii. Example: Use 10 μL of 10 mM methylsuccinate, 8 mL of HPLC-grade methanol, and 1.99 mL of HPLC-grade H_2_O to make 10 mL of extraction buffer.
    iii. Cool the extraction buffer on dry ice for a few minutes to equilibrate.
      ***Note:*** Make the extraction buffer fresh and keep it chilled. If left over, it can be stored at 4°C and used within a week.
      ***Note:*** Methylsuccinate is used here as an internal standard, which accounts for differences in how well metabolites are extracted from each sample. It can be substituted with or used alongside norleucine and norvaline. Amino acids that are both 15N- and uniformly 13C-labeled can also be used.
      **CRITICAL:** Methanol is highly flammable and toxic if swallowed, inhaled, or absorbed through skin. It can cause organ damage, including blindness. Handle in a chemical hood with proper PPE (lab coat, gloves, and safety goggles) – Refer to the SDS.
      **CRITICAL:** Use an ice-cold extraction buffer and acclimate the pipette tip to the cold mix before adding to ensure minimal pipetting error. After adding the extraction buffer, keep samples and standards on dry ice until homogenization. Sonicate the samples within an hour of adding the buffer to minimize metabolite degradation until further processing.

7. Prepare Methoxyamine-HCL solution in pyridine.
  a. Make 20 mg/mL methoxyamine-HCL in pyridine.
    i. Prepare 10 μl of the solution for each sample.
    ii. Example: Add 10 mg methoxyamine-HCL in 500 μl pyridine.
    iii. Mix the solution using a rotating mixer until the solid is dissolved.

***Note:*** Prepare it immediately before use. Do not store or reuse.

**CRITICAL:** Methoxyamine HCl is an irritant and harmful if exposed. Handle in a chemical hood with proper PPE (lab coat, gloves, and safety goggles) - Refer to the SDS.

**CRITICAL:** Pyridine is flammable, volatile, corrosive, and irritant and harmful if exposed. Handle in a chemical hood with proper PPE (lab coat, gloves, and safety goggles) - Refer to the SDS.

8. Generate a selected ion monitoring (SIM) mass spectrometry method using standards to verify retention times and masses.^2^

***Note:***Table 1 lists the retention times and masses we track for routine analysis. Chromatography type (GC vs. LC), column length, and sample preparation (derivatized vs. underivatized) will alter these parameters.

9. Ensure all necessary software is downloaded and can be used.^2^ For our routine analysis of data, we use MSD Chemstation (Agilent Technologies, Inc.) and Isocor (v1.0).

### Protein quantification

**Timing: 15–20 min**

10. Prepare RadioImmunoPrecipitation Assay (RIPA) buffer.
  a. Prepare at least 150 μL of buffer for sample.
  b. Example: In 6.4 ml of milli-Q H2O, add 1.5 mL of 1M NaCl, 1 mL of 10% Triton-X, 500 μL of 10% Sodium deoxycholate, 100 μL of 10% SDS, 500 μL of Tris-HCL (pH 8) and add 10 μL of 100X protease inhibitor to prepare 10 mL RIPA buffer.

***Note:*** Add the protease inhibitor immediately before use. Keep the buffer cold in ice. The buffer without protease inhibitor can be stored at 4°C for a month.

## Key resources table

**Table:** 

REAGENT or RESOURCE	SOURCE	IDENTIFIER
**Chemicals, peptides, and recombinant proteins**		

D-Glucose (U-^13^C_6_, 99%)	Cambridge Isotope Laboratories, Inc.	Catalog No.: CLM-1396-PK
D-Glucose (U-^12^C, 99.9%)	Cambridge Isotope Laboratories, Inc.	Catalog No.: CLM-4819-PK
HBSS	Thermo Fisher Scientific	Catalog No.: 14-175-095
NaCl	Fisher Chemical	Catalog No.: S271-500
KCl	Sigma-Aldrich	CAS No.: 7447-40-7
KH_2_PO_4_	Sigma-Aldrich	CAS No.: 7778-77-0
MgSO_4_ · 7H_2_O	Fisher Chemical	Catalog No.: 01-337-186
HEPES	Sigma-Aldrich	CAS No.: 7365-45-9
CaCl · 2H_2_O	Sigma-Aldrich	CAS No.: 10035-04-8
NaHCO_3_	Fisher Scientific Company	Catalog No.: BP328-500
Methanol	Sigma-Aldrich	CAS No.: 67-56-1
Methylsuccinate	Sigma-Aldrich	Catalog No.: M81209 CAS No.: 498-21-5
Methoxyamine HCl	Sigma-Aldrich	Catalog No.: 226904 CAS No.: 593-56-6
Pyridine	Sigma-Aldrich	Catalog No.: 270970 CAS No.:110-86-1
TBDMS	Sigma-Aldrich	Catalog No.: 394882 CAS No.:77377-52-7
HPLC-grade H_2_O	Sigma-Aldrich	Catalog No.: 270733 CAS No.: 7732-18-5

**Critical commercial assays**		

BCA protein concentration assay	Thermo Fisher	Catalog No.: 23225

**Experimental models: Organisms/strains**		

Mouse: C3;CAnN-Pde6b^atrd1^/H	EMMA	Catalog No.: EM:01292; RRID:IMSR_EM:01292
Mouse: B6J.Rho^C110R/+^	Tsang Laboratory	N/A
Mouse: B6J.Pde6γ^CreERT2^	Tsang Laboratory	N/A

**Software and algorithms**		

MSD Chemstation	Agilent Technologies	Version: E.02.01.1177
IsoCor	Millard et al., 2012	Version: v1.0
Gen5	Agilent Technologies	Part# 7160204 Version: 1.12

**Other**		

0.22 μm filter	Fisher Scientific	Catalog No.: SLGPR33RS
Petri Dishes with Clear Lid	Fisher Scientific	Catalog No.: FB0875712
Corning PYREX Round Media Storage Bottles	Fisher Scientific	Catalog No.: 10-462-716
24-well plate	Corning Costar	Catalog No.: 09-761-146
96-well plate	Corning Costar	Catalog No.: 07-200-90
Cohan-Vannas Spring Scissors	Fine Scientific Tools	Catalog No.: 15000-01
Forceps	Fine Scientific Tools	Catalog No.: 15000-02
Fine Forceps	Fine Scientific Tools	Catalog No.: 11274-20
GC-MS autosampler vials	Agilent Technologies	Catalog No.: 5190-9589
GC-MS autosampler vial screw-caps	Agilent Technologies	Catalog No.: 5190-3156
GC-MS vial inserts	Agilent Technologies	Catalog No.: 5183-2085
Olympus	Stereo Microscope With Two Objectives	SKU: SZX10
Centrifuge	Sorvall Legend Micro 17, Thermo Fisher Scientific	Catalog No.: 75-002-431
Probe sonicator	Branson Sonifier 250, VWR	Catalog No.: 10820-440
Vacuum concentrator	Speed-Vac SVC100, Savant	Model No.: SVC-100
Lyophilizer	FreeZone 4.5 Plus, Labconco, Fisher	Labconco™ 7386041 Fisher Catalog No.:16-208-501
Vacuum pump	1402N, Welch ChemStar	Catalog No.: 1402N
Incubator	Isotemp, Fisher Scientific	Catalog No.: 11-690-650D
Harris	−80°C Ultra Low Temperature Cryogenic Freezer	Model No.: DLT-21V-85D12
Plate reader	BioTek Synergy 4 plate reader (Agilent Technologies)	Model No.: S4MLFPTA
Gas Chromatograph-mass spectrometer (GC-MS) Autosampler	Agilent Technologies	Model No.: 7693
GC-MS Chromatograph	Agilent Technologies	Model No.: 7890A
GC-MS mass spectrometer	Agilent Technologies	Model No.: 5975C
Heat block	Reacti-Therm III #TS-18824, Thermo Fisher Scientific	Catalog No.: TS-18824

## Materials and equipment

**Table:** KRB Buffer

Reagent	Final concentration	Amount
NaCl	98.5 mM	2.998 g
KCl	4.9 mM	0.1905 g
KH2PO4	1.2 mM	0.085 g
MgSO4 · 7H2O	1.2 mM	0.154 g
HEPES	20 mM	2.4825 g
CaCl · 2H2O	2.6 mM	0.199 g
NaHCO3	25.9 mM	1.1335 g
Milli-Q 2H2O	N/A	500 ml

Store at 4°C for up to 24 months.

**Table:** RIPA Buffer

Compound	Stock concentration	Final concentration	Volume needed per 10 mL
Sodium Chloride	1 M	150 mM	1.5 mL
Triton X	10%	1%	1 mL
Sodium Deoxycholate	10%	0.5 %	500 μL
SDS	10%	0.1%	100 μL
Tris-HCl (pH 8)	1 M	50 mM	500 μL
H_2_O	N/A	N/A	6.4 mL
Protease inhibitor cocktail	100X	1X	10 μL

Stored at 4°C for a month.

## Step-by-step method details

### Incubation of retinal tissue in isotopically labeled glucose

**Timing: 20–25 min (per eyeball; can be overlapped during incubation)**

This protocol enables the labeling and tracking of TCA and glycolytic cycle intermediates using U^13^C_6_-glucose for metabolic flux analysis. The method allows simultaneous processing of tissue.

***Note:*** Keep track of which tissue came from which mouse as it will be necessary for step 2.d and making a metric graph in the data analysis section.

1. Obtain mouse tissue of interest (RPE/choroid or neuroretina).
  a. Euthanize the mouse by awake cervical dislocation.
  b. Enucleate both eyes and place in a clean petri dish of room temperature with 5 ml HBSS.
  c. Dissect retina and RPE-choroid tissues.^3^
    i. Using the Cohan-Vannas spring scissors, forceps and fine forceps, trim as much fat, connective tissue, and muscle from around the eyeball as possible

**CRITICAL:** Don't cut through the eyecup or the neuroretina when handling the tissues, especially after detaching the retina from the eyecup.

**CRITICAL:** Carefully grasp near the base of the optic nerve to minimize the amount of connective tissue on the sample before dissecting.

***Note:*** A dissection plate can be placed on top of ice to maintain a cold temperature and better preserve tissue and cellular integrity. This, however, could introduce thermal shock to the tissue after incubation at 37°C. Adjust this step according to how the tissue responds during experimental runs.

2. Wash and incubate the tissue.
  a. Wash the tissue in HBSS (quick dip and swirl).
  b. Incubate the tissue in the 12C well for 15 minutes. Keep the plate in the incubator (37°C, 5% CO2) during this time.
  c. Wash the tissue in HBSS.
  d. Incubate the tissue in the 13C well for 30 or 90 seconds, depending on the experimental group. Keep the plate in the incubator during this incubation time (37°C, 5% CO2).
  e. Wash the tissue in HBSS.
  f. Repeat steps 2.a to 2.e for each tissue sample.

***Note:*** During the 15-minute period in step 2.b, another eyeball or tissue of interest could be processed from the same or a different mouse to stagger experimental samples.

***Note:*** Consider separating eyes from each animal into different incubation time groups for higher experimental rigor.

***Note:*** 30 and 90-s incubation times in step 2.d work well for some experimental purposes (for example, 13C labeling of glycolytic intermediates) but less well for others (13C labeling of TCA cycle intermediates). These times were determined to capture maximal flux of the glycolytic pathway’s turnover of 13C6 metabolites. Shorter time frames could limit 13C intake, and longer time frames could cause saturation of most metabolites, making their comparison between groups difficult. Adapt incubation times and tracers to the needs of the experiment.

**CRITICAL:** When processing several tissue samples, prioritize the timing of the labeled incubations over non-labeled normalization incubations (13C over 12C, respectively).

**CRITICAL:** Do not use the same well for HBSS washing or incubating twice, especially after 12C and 13C incubation. All HBSS wells should be kept at room temperature when not in use to prevent thermal shock to the tissue.

3. At the end of the post 30 or 90-second HBSS wash in step 2.e, place the sample into a 1.5 mL Eppendorf tube and snap-freeze the tube in liquid nitrogen.
  a. Rinse forceps before subsequent dissections to prevent contamination between samples.
  b. Snap-frozen tissue can be stored at −80°C until further processing.

**Pause Point:** A break in sample processing may be taken, depending on available time and equipment. Samples can be stored in −80°C for at least 12 months.

### Metabolite extraction

**Timing: 4–5 h**

This process separates metabolites from cellular debris, lipids, and proteins while minimizing the activity of enzymes that could alter the amounts of particular metabolites. This procedure was adapted from Du et al.^2^

**CRITICAL:** Prior to the experiment, prepare a standard mix containing 50 μM of metabolites (see Table 1). This solution can be dried and kept in that state. Store single-use tubes at −80°C to maximize the stability of the metabolite standards.

4. Thaw the standard mix and samples in dry ice.
5. Add 150 μL of ice cold extraction buffer to each sample and prepared standard curve dilutions.

***Note:*** Keep the standards and samples cold in dry ice.

6. Sample preparation.
  a. Homogenize samples using a probe sonicator.
    i. Sonicate 10 cycles at power 2 and 90% duty cycle for RPE/Choroid.
  b. Precipitate protein by incubating sample tubes on dry ice for 45 minutes.
  c. Pellet protein and cellular debris by centrifuging at 17,000 x g, 30 minutes, 4°C.
    i. Transfer the supernatant to a new tube.
  d. Dry the supernatant using the SpeedVac connected to lyophilizer.
  e. Place dried samples and standard dilutions in the −80°C freezer until further processing.

**CRITICAL:** Use equivalent settings for all samples of a given tissue type.

***Note:*** The supernatant in step 6.c contains metabolites, and the pellet contains proteins.

***Note:*** The pellet in step 6.c.i can be dried and later solubilized in a RIPA buffer (KRT) to assess protein content or composition.

***Note:*** Ensure the supernatant in 6.d is completely dried. Usually it takes 2–3 hours.

***Note:*** Due to the dense extracellular matrix and structural proteins, RPE-choroid-sclera is difficult to homogenize with a probe sonicator alone. Other methods can be used to ensure complete homogenization, but when sonicating RPE-choroid, pigmented RPE quickly disburses throughout the extraction buffer, indicating RPE cell homogenization.

### Metabolite derivatization

**Timing: 4–5 h**

This process adds chemicals that modify metabolites. These modifications prevent the formation of hydrogen bonds, and as a consequence, molecules are made more volatile. Ketones and aldehydes are derivatized by methoxyamine dissolved in pyridine. Amines, carboxylic acids, alcohols, and amides are derivatized bytert-butyl-dimethylsilyl trifluoromethanesulfonate (TBDMS). This procedure was adapted from Du et al.^2^

**CRITICAL:** Perform all the steps below in a chemical hood.

7. Chemical derivatization.
  a. Remove the dried samples and standards from the −80°C freezer.
  b. Further dry the supernatant collected from the extraction process for 30 minutes.
  c. Add 10 μL freshly prepared methoxyamine solution to each dried sample and standard dilution.
    i. Vortex and spin down each tube briefly.
    ii. Incubate at 37°C for 1.5 hours.
  d. Add 10 μL of TBDMS solution to each sample and standard dilution.
    i. Vortex and spin down each tube briefly.
    ii. Incubate at 70°C for 1 hour.
8. GC-MS sample preparation.
  a. Centrifuge samples at 17,000 x g for 1 minute at room temperature to collect all the liquid at the bottom of the tube.
  b. Place glass inserts in autosampler vials.
  c. Transfer the solution containing derivatized metabolites from the Eppendorf tubes to the glass inserts.
  d. Cap the vials and run the samples in GC-MS.

**CRITICAL:** Methoxyamine-HCl is hygroscopic. Minimize the time exposed to the atmosphere, and store with a desiccant.

### Gas chromatography-mass spectrometry

**Timing: 2–3 days, depending on the number of samples**

This process outlines the steps for setting up the GC-MS instrument, running the samples in the instrument, and analyzing the data acquired from the run. This procedure was adapted from Du et al.^2^

***Note:*** This protocol relies on the equipment listed in the KRT.

9. Hardware and software preparation.
  a. Prepare the sequence file.
  b. Place standard and sample vials on the autosampler and run the samples.
  c. Add fresh wash solvent for the injection needle.
    i. Discard any liquid waste found in needle wash waste vials.
  d. Load the sequence file containing the order in which samples will be injected.
  e. Run the sequence file (depends on the sample type, method, and instrument used).
    i. Example: The temperature gradient begins at 100°C with a hold time of 4 min and increases to 300°C at a rate of 5°C/minute with 5 minutes hold time.
    ii. Example: The temperatures reset: 250°C for the inlet, 280°C for the transfer line, 230°C for the ion source, and 150°C for the quadrupole.

**CRITICAL:** For step 9.b, ensure that the helium carrier gas has the secondary pressure gauge at ∼40 psi; otherwise, it could run out in the middle of the run.

**CRITICAL:** For step 9.c, ensure solvent is replaced every ∼24 h per solvent wash vial.

***Note:*** The instrument has a solvent delay, which varies in time for each machine. Do not skip the solvent delay to help lengthen the instrument's lifespan.

**Figure 1:**
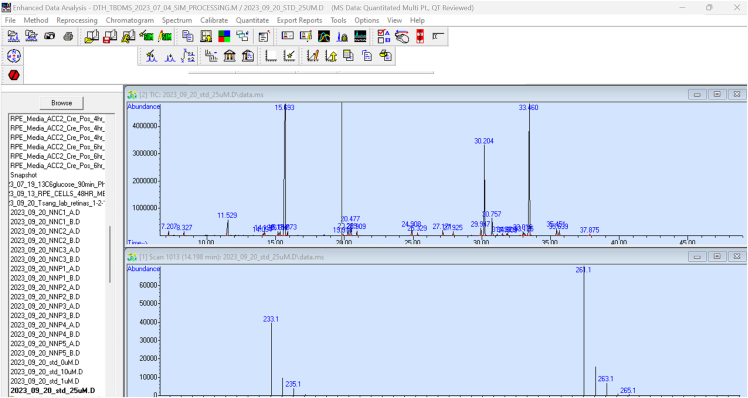
Standard Layout of MSD ChemStation for 25μM sample data
  c. Retrieve and modify the prepared quantification method to allow the program to recognize metabolite peaks at pre-programmed retention times.
    i. Retrieve the quantification method from the saved files.
    ii. Example: The method for quantification is usually located within msdchem -> 1 -> method. Active quantitation methods are bolded in the file browser.
    iii. Review the peak recognition for each molecule and isotopologue to ensure optimal results. Review and correct the area under the curve by clicking on the ‘QEdit’ icon or typing “QEdit“ in the search bar and pressing “enter” (Figure 2).

10. Quantify relative metabolite abundance.
  a. Acquire the sample data and copy the data into a path that is recognized by the analysis software.
    i. Example: Paste data into the following folder: OS (C:) -> msdchem -> 1 -> data.
  b. Open MSD ChemStation program and load the data by double-clicking the file name (Figure 1).

**Figure 2:**
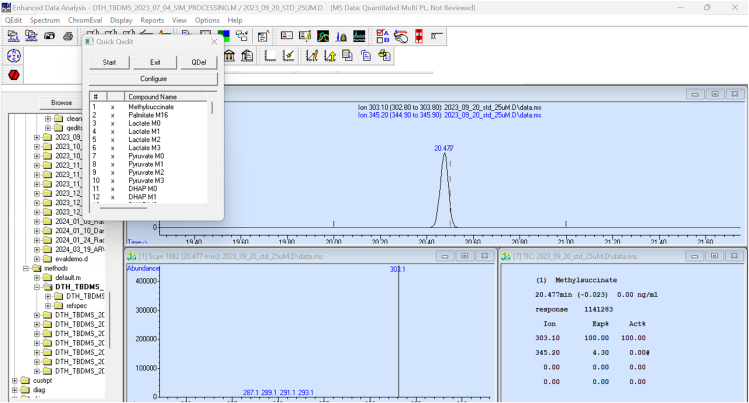
MSD ChemStation Qedit window
    iv. Adjust the red line underneath each metabolite ‘peak’ to ensure correct integration (Figure 3).

**Figure 3:**
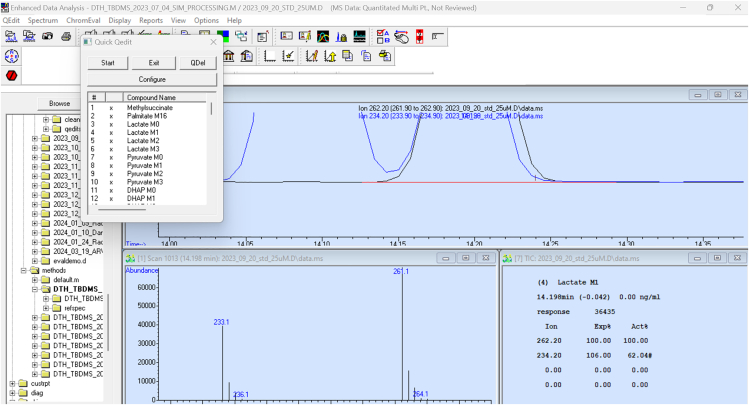
Integrated peak for lactate sample
    v. Exit on the Qedit window and save any changes and reviewed files.
    vi. Export the reviewed results.
    vii. Correct for natural isotopic abundance using IsoCor.^4^

***Note:*** This protocol uses MSD ChemStation E.02.01.1177 to quantify our data.

***Note:*** A report could be exported to Excel by clicking on the “Export Reports” tab and then clicking on “Quantitation Results Report to XLS” (Figures 4 and 5).

***Note:*** For step 10.c, the retention time might shift over a period of months. The retention times expected by the software should be modified to reflect the retention times of known standards.

***Note:*** For the step 10.c.i.2, on a given chromatogram, the dotted vertical line on the chromatogram represents the expected retention time.

11. Data integration into molar amounts of distinct isotopologues.
  a. Copy the data for each standard and sample (intensity and the fractional labeling of isotopologues) to a data analysis software.
  b. Normalize the abundance of each metabolite to the abundance of the internal standard.
  c. Sum the abundance of all isotopologues of a given metabolite.
  d. Using a linear function defined by the slope and intercept of the standard curve, translate signals acquired by the mass spectrometer into molar amounts of each metabolite.
  e. Multiply the molar amount of a given metabolite by the fractional labeling of each isotopologue to generate the amount of a given isotopologue.
  f. If protein amounts were quantified, normalize the amount of a metabolite in a given sample to the amount of protein in the same sample.

**Figure 4:**
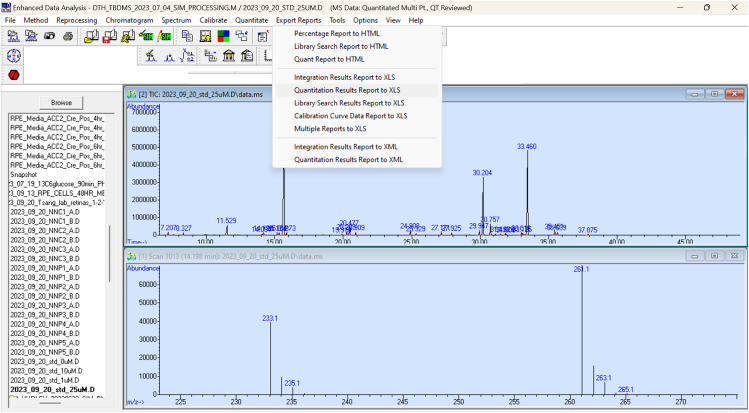
Excel export pathway for MSD ChemStation

**Figure 5:**
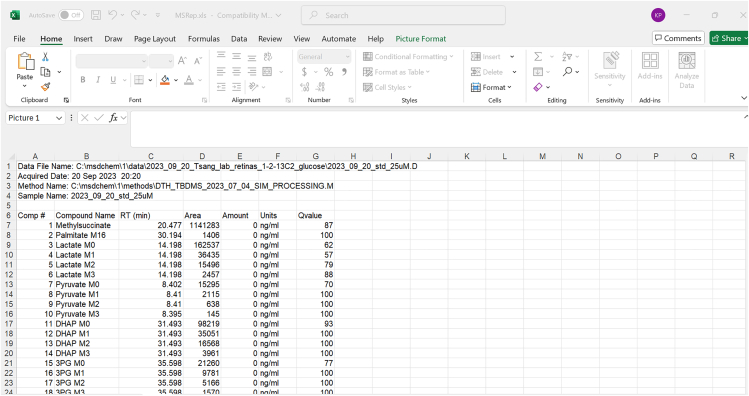
Exported Excel sample data

### Protein quantification

**Timing: 60–90 min**

This process quantifies the total protein content from the remaining tissue pellet after the metabolite extraction.

12. Sample preparation.
  a. Add 150 μL of RIPA lysis buffer to each tissue pellet generated during the “metabolite extraction” procedure, step 2.d.
  b. Disrupt tissue pellet with an ultrasonic homogenizer.
    i. Sonicate 10 cycles at power 2 and 90% duty cycle for RPE/Choroid.
  c. Centrifuge to pellet large debris at 17,000 x g at 4°C for at least 5 minutes.
  d. Add 10 μL of debris-free supernatant to a well of a clear-bottom 96-well plate.

***Note:*** Protein concentration assays perform best when samples and standards are assessed in duplicate or triplicate. Standards are known concentrations of bovine serum albumin. When assessing 10 μL of retinal extract, protein standard values ranging from 1 - 20 μg / well work well.

13. Perform a protein concentration assay using instructions provided by the assay manufacturer.

***Note:*** BCA assay was used for this protocol.

## Expected outcomes

This protocol yields information on the metabolic flux of various metabolites associated with the labeled compound and pathway of interest. We have designed the experiment to probe glycolytic flux using 13C-labeled glucose. This information is useful for understanding the overall health and energy production capabilities of biological tissues. In inherited retinal dystrophies such as retinitis pigmentosa, phenocopied by the mice chosen in our experiment,^1^ glycolytic flux impairment is implicated as a direct consequence of the underlying mutations and is thought to directly contribute towards overall photoreceptor degeneration, cell death, and blindness.^5^ Gauging how causative underlying mutations impact this process will inform the mechanism of degeneration in RP and potentially other neurodegenerative disorders such as Parkinson’s or Alzheimer’s disease. The direct outcome of this protocol is the concentrations vs. time of labeled metabolites associated with glycolysis and subsequent TCA cycle function. The outcome of this protocol reveals changes in the TCA cycle and glycolytic function in degenerate retinas and controls. In our case, glycolytic flux markers have shown an increase, and TCA cycle intermediates showed a decrease as a result of our genetic modulation, as seen in Nolan et al.^1^ This information will be essential in evaluating therapeutics for years to come and is a direct window into cellular energy metabolism, maintenance, and health, critical parameters for future precision or gene-agnostic therapies.

## Limitations

While metabolite tracing provides insight into the TCA cycle of the cell population, it offers limited information on overall cell health, offshoots, or other tangentially related metabolic pathways, or differences in the transcript and protein levels of enzymes not directly associated with the direct target of interest. The current protocol is not optimized for long-term incubation studies with labeled metabolites, such as 20 or 40 minutes. Another shortsightedness of this protocol manuscript is the lack of consideration of the composition and concentration of metabolic intermediates in KRB, which do not match those of circulation or interstitial fluid. Because some of these factors may interact with and affect glucose metabolism, their absence in our experiments may prevent an accurate assessment of physiological metabolic rates from glucose. Similarly, inner retinal blood supply is unavailable in an *ex vivo* retina preparation. This prevents the appropriate delivery of glucose, oxygen, and other crucial intermediates. Thus, metabolic rates obtained ex vivo might exclude activities from some cell populations. The function of retinal tissues may be considerably higher *in vivo* compared to our *ex vivo* incubations. Submerging the retina or RPE in stable, high glucose concentrations may not accurately reflect the typical fluctuating metabolomics of TCA cycle intermediates. An additional limitation is that *in vivo* nutrient flow through the retina is severely impacted by apical and basolateral blood-retinal barrier filtering. This aspect is absent from *in vitro* metabolic tracing experiments. Lastly, the TCA cycle is not fed solely by glucose and has other inputs from metabolic processes. This introduces ample space for an incorrect analysis or interpretation of results.

## Troubleshooting

### Problem

Sometimes the tissue can become slushy and get severely damaged during transfer from one well to another. Degraded, damaged, or degenerated tissue may fall into pieces or behave as a non-uniform sample, referring to step 2 of «Incubation of retinal tissue in isotopically labeled glucose».

### Potential solution

Instead of using a pipette, try carefully scooping the tissue with a slotted spoon. While this may not fix the damage to the tissue, it may help prevent further damage from occurring, keeping the sample in one piece/place.

### Problem

Dissection is a difficult procedure and takes many attempts to perfect. Because of this, damage to the RPE or retina layer could happen very easily, referring to step 1 of «Incubation of retinal tissue in isotopically labeled glucose».

### Potential solution

If you notice a severe mixture of the RPE and the retina layers or damage to the tissue, causing it to fall apart into several segments, discard the sample as a whole. Discarding the sample at this stage is better than obtaining and analyzing suboptimal data.

## Resource availability

### Lead contact

Further information and requests for resources and reagents should be directed to and will be fulfilled by the lead contact, Stephen H Tsang (sht2@cumc.columbia.edu).

### Technical contact

Questions about the technical specifics of performing the protocol should be directed to and will be answered by the technical contact, Georgy Komissarov (gk2643@cumc.columbia.edu).

### Materials availability

This study did not generate new unique reagents.

### Data and code availability

All data reported in this paper will be shared by the lead contact, Stephen H. Tsang (sht2@cumc.columbia.edu) upon request. Any non-publicly available datasets can be obtained through the lead contact, Stephen H Tsang (sht2@cumc.columbia.edu) upon request. This paper does not report original code. Any additional information required to reanalyze the data reported in this paper is available from the lead contact, Stephen H. Tsang (sht2@cumc.columbia.edu) upon request.

## Acknowledgments

We thank Prof. Guo-Hua Fong, Siyuan Liu, Yong-Shi Li, Salvatore M. Caruso, Marilyn Rodriguez, John Peregrin, Jimmy Duong, Li-Juan Duan, Anders Knudsen, and members of Jonas Children’s Vision Care (JCVC) for sharing mice and ideas; the JCVC is supported by the National Institutes of Health U01 EY030580, U54OD020351, R24EY028758, R24EY027285, 5P30EY019007, R01EY018213, R01EY024698, R01EY033770, R21AG050437, F31EY033660, NYEE Foundation, the Foundation Fighting Blindness TA-GT-0321-0802-COLU-TRAP, Lynette & Richard Jaffe Foundation, Nancy & Kobi Karp, the Crowley Family Funds, the Rosenbaum Family Foundation, Alcon Research Institute, the Gebroe Family Foundation, the Piyada Phanahat fund, and Research to Prevent Blindness (RPB) Physician-Scientist Award, unrestricted funds from RPB, New York, NY, USA. The funding organizations had no role in the design or conduct of this research. BioRender.com was used to create the graphical abstract.

## Author contributions

Conceptualization, N.D.N., D.T.H., J.B.H., and S.H.T.; methodology, G.K., K.P., N.D.N., T.W., D.T.H., A.D., and B.M.R.; investigation, G.K., K.P., N.D.N., and D.T.H.; writing – original draft, G.K., K.P., N.D.N., T.W., and D.T.H.; writing – review and editing, G.K., K.P., N.D.N., T.W., D.T.H., J.B.H., and S.H.T.; funding acquisition, N.D.N., D.T.H., A.D., J.B.H., and S.H.T.; supervision, N.D.N., D.T.H., J.B.H., and S.H.T.

## Declaration of interests

S.H.T. receives research support from Emendo. He is on the scientific and clinical advisory board for Emendo, Medical Excellence Capital, and Nanoscope Therapeutics. S.H.T. holds a patent related to this work: WO2018232227A1.
